# Reduced placental volume and flow in severe growth restricted fetuses

**DOI:** 10.6061/clinics/2016(06)08

**Published:** 2016-06

**Authors:** Renata Montes Dourado Abulé, Lisandra Stein Bernardes, Giovana Farina Doro, Seizo Miyadahira, Rossana Pulcinelli Vieira Francisco

**Affiliations:** Faculdade de Medicina da Universidade de São Paulo, Departamento de Obstetrícia e Ginecologia, Disciplina de Obstetrícia, São Paulo/SP, Brazil

**Keywords:** Placenta, Ultrasonography Doppler, Imaging Three-Dimensional, Placenta Circulation, Fetal Growth Retardation

## Abstract

**OBJECTIVES:**

To evaluate placental volume and vascular indices in pregnancies with severe fetal growth restriction and determine their correlations to normal reference ranges and Doppler velocimetry results of uterine and umbilical arteries.

**METHODS:**

Twenty-seven fetuses with estimated weights below the 3^rd^ percentile for gestational age were evaluated. Placental volume and vascular indices, including vascularization, flow, and vascularization flow indices, were measured by three-dimensional ultrasound using a rotational technique and compared to a previously described nomogram. The observed-to-expected placental volume ratio for gestational age and observed-to-expected placental volume ratio for fetal weight were calculated. Placental parameters correlated with the Doppler velocimetry results of uterine and umbilical arteries.

**RESULTS:**

The mean uterine artery pulsatility index was negatively correlated with the observed-to-expected placental volume ratio for gestational age, vascularization index and vascularization flow index. The observed-to-expected placental volume ratio for gestational age and observed-to-expected placental volume ratio for fetal weight and vascularization index were significantly lower in the group with a bilateral protodiastolic notch. No placental parameter correlated with the umbilical artery pulsatility index.

**CONCLUSIONS:**

Pregnancies complicated by severe fetal growth restriction are associated with reduced placental volume and vascularization. These findings are related to changes in uterine artery Doppler velocimetry. Future studies on managing severe fetal growth restriction should focus on combined results of placental three-dimensional ultrasound and Doppler studies of uterine arteries.

## INTRODUCTION

Fetal growth restriction (FGR) is a major complication of pregnancy and is associated with high rates of perinatal morbidity and mortality. The frequency of FGR-associated neonatal adverse outcomes is directly related to the severity of FGR, with most cases of poor prognosis associated with fetal weights below the 3^rd^ percentile 1-3. Abnormal placental development and decreased uteroplacental perfusion are associated with pregnancy complications and are considered possible causes for FGR 4.

Currently, it is possible to evaluate both placental volume and vascularization using three-dimensional ultrasound (3DUS) 5-8. Few studies have investigated the importance of this technique in the assessment of FGR. Three studies have evaluated the placental vascular indices of growth-restricted fetuses using the vascular placental biopsy technique described by Mercé 9,10, in which isolated spherical samples of the placenta are analyzed. These studies reported decreased placental vascular indices in growth-restricted fetuses 11-13. Similarly, two additional studies assessed the placentas of growth-restricted fetuses using the Virtual Organ Computed-Aided Analysis (VOCAL) rotational technique, which allows the measurement of vascular indices across the entire placental volume. The evaluation of entire placentas revealed that FGR is correlated with decreases in placental volume and vascular indices 14,15. All these previous studies focused on moderate FGR (below the 10^th^ percentile) in low-risk patients 11-15.

The most severe cases of FGR have fetal estimated weights below the 3^rd^ percentile 1-3. In these subjects, changes in placental volume and vasculature may be correlated with Doppler parameters recorded for the fetus and mother. However, there are currently no published studies that have investigated this correlation in severe growth-restricted fetuses. Because pregnancy follow-up may be challenging in more severe cases, studies specifically focusing on this group of fetuses may aid clinical decision-making processes in the future. Therefore, the present study aimed to evaluate placental volume and vascular indices in severe growth-restricted fetuses and to determine whether correlations exist between these findings and the results of maternal and fetal two-dimensional (2D) Doppler imaging.

## MATERIALS AND METHODS

### Patients and study design

This study was conducted at the Hospital das Clínicas da Faculdade de Medicina da Universidade de São Paulo, Disciplina de Obstetrícia (HCFMUSP). It was a descriptive study where correlations were analyzed between placental volume and blood flow among pregnancies with severe FGR.

The following inclusion criteria were applied: estimated fetal weight (as measured by ultrasound) below the 3^rd^ percentile of the Hadlock table 16, singleton pregnancy, normal fetal morphological ultrasound evaluation and reliable pregnancy dating established by the date of the last menstrual period and by sonography measurement of the crown-rump length during the first trimester. The following exclusion criteria were applied: inability to visualize the entire placenta, abnormal karyotype, diagnosis of fetal malformations and infection of the fetus.

All ultrasound evaluations were performed by the same observer (RMDA). Fetal weight, placental volume and vascularization, and Doppler velocimetric parameters were evaluated. Measurements were performed using a Voluson 730 Expert™ ultrasound machine (GE Healthcare, Milwaukee, WI, USA) equipped with a 1–5 MHz convex transducer and a 4–8 MHz transducer for 3D assessment of placental volume. The estimated fetal weight was calculated using the biparietal diameter, head and abdominal circumferences, and fetal femur length 17. Fetal weight percentile was calculated using the Hadlock formula 16.

### Assessment of 2D Doppler velocimetric parameters

To ensure that suitable sonograms were obtained, we used color Doppler mapping to identify blood vessels and all recordings were performed in the absence of maternal or fetal movement. Sonograms were considered adequate when three consecutive waves of similar flow rates were obtained. To standardize our umbilical artery (UA) evaluation methodology, evaluations were made on the segment proximal to the placental insertion 18. After obtaining the sonogram, the pulsatility index (PI) was calculated 19. The uterine arteries (UtAs) were evaluated in the crossing of the external iliac vessels. After a sonogram was recorded, the PI was calculated for both UtAs and the mean PI was then calculated as a simple average of the values obtained for the right and left UtAs. UtAs were also classified according to the absence or presence of a bilateral diastolic notching 20.

### Assessment of placental volume and vascular indices

3DUS placental evaluations were conducted using real-time scanning with a 4–8 MHz transducer. The measurement parameters and specific techniques used were previously described 6,7. To standardize the acquisition procedures used to obtain 3D placental vascular indices, the settings for power Doppler were as follows: angio mode, cent; smooth, 4/5; FRQ, low; quality, 16; density, 6; enhance, 16; balance, GO150; filter, 2; actual power, 2 dB; and pulse repetition frequency, 0.9. Images were captured using the lowest gain possible, allowing us to obtain a proper image of the placental vascular tree while avoiding artifacts. The entire placental view was generated using 2D ultrasound. For posterior and laterally located placental segments, the transducer was held at a slight lateral inclination to acquire better images. Placental distance from the probe was also recorded. To minimize any interference from acoustic shadowing due to fetal positioning, the transducer was slightly inclined and a higher sweep angle 90° was used to include the largest longitudinal axis of the placenta. Images affected by this movement (maternal, fetal, or transducer) were discarded and recaptured. After visualization of the placental vascular tree, 3D static power Doppler scanning was performed. After scanning the region of interest, placental volume was measured using the VOCAL rotational technique and VOCAL™ software (3D SonoView, GE Medical Systems, Milwaukee, WI, USA). This technique comprises repeatedly outlining the contour of the placenta after rotating the placental image six times in 30° increments. After completing a full rotation, another analysis was performed using VOCAL™ software (3D SonoView, GE Medical Systems, Milwaukee, WI, USA), which automatically calculates placental volume, vascularization index (VI), flow index (FI) and vascularization flow index (VFI).

### Statistical analysis

Placental volume and vascular indices (VI, FI and VFI) were classified according to De Paula et al. 6,7 as reduced (below the 10^th^ percentile) and normal reference ranges.

Because placental volumes vary according to gestational age and fetal weight, the observed values were compared with the expected values (50^th^ percentile) for the appropriate gestational age and fetal weight. The observed-to-expected placental volume ratio at gestational age (OEPVR-GA) and observed-to-expected placental volume ratio to fetal weight (OEPVR-FW) were calculated. Placental parameters were then correlated with the mean PI for the UtAs and with the PIs for the UAs. The placental parameters were also evaluated according to the presence of bilateral protodiastolic notching in the UtAs.

The potential correlation of placental 3DUS parameters to 2D Doppler findings was evaluated using Spearman’s correlation coefficient for nonparametric variables and the Pearson correlation coefficient for FI, which was the only parametric variable. Placental parameters were compared according to the presence or absence of bilateral UtA diastolic notches using the Mann–Whitney test for nonparametric variables and Student’s t-test for FI. A *p*-value of less than 0.05 was considered statistically significant. All statistical analyses were performed using SPSS Statistics for Windows, Version 17.0 (SPSS Inc, Chicago, USA).

## ETHICS

All subjects provided written informed consent to participate in the study, which was performed with approval from the Institutional Review Board of the Faculdade de Medicina da Universidade de São Paulo (protocol number 0766-10).

## RESULTS

In total, 30 patients were evaluated. Three patients (10%) were excluded because it was not possible to obtain a 3DUS evaluation of the entire placenta. Maternal and fetal characteristics are presented in Table 1.

### Placental volume and vascular index analyses

Fetal placental volume and vascular indices are presented in Table 2.

Correlation analyses evaluating placental parameters and gestational age are presented in Table 3. The only parameter that showed a significant correlation with gestational age was placental volume.

All placental parameters were classified as either normal or reduced. The results of these analyses are presented in Table 4. The frequency of reduced placental volume and vascularization in the severe FGR cases were higher than those observed in the general population.

### Correlation of placental parameters to 2D Doppler findings

The results of correlation analyses between placental parameters and Doppler velocimetry results of uterine and umbilical arteries are presented in Table 5. The mean UtA PI significantly correlated with OEPVR-GA, VI and VFI. No placental parameters correlated with UA PI.

Placental parameters were evaluated according to the presence or absence of a bilateral UtA diastolic notch and the results are presented in Table 6. OEPVR-GA, OEPVR-FW and VI were significantly lower in the group with a bilateral UtA diastolic notch. The mean gestational age of the patients with a bilateral UtA diastolic notch was 30.04 (±4.1) weeks, while the mean gestational age of the pregnant women with no bilateral UtA diastolic notch was 32.2 (±3.7) weeks (*p*=0.156).

## DISCUSSION

This study observed, for the first time, an inverse correlation between placental VI and volume and uterine resistance in severe growth-restricted fetuses. Similarly to previous reports 13-15, all placental vascular indices, including volume, were reduced when compared to normal-growth fetuses.

### Relationship between placental parameters and 2D Doppler of UtAs

Placental volume was inversely correlated with UtA resistance, demonstrating that smaller placentas correlate to greater reductions in blood flow. Although no reported studies have investigated this correlation in FGR fetuses, decreased placental volume in the first trimester has been related to abnormal UtA Doppler findings in the prediction of preeclampsia and FGR 21,22. All the above findings corroborate the hypothesis that placental volume is related to the degree of impaired development of the placental vascular tree, which may be the primary underlying cause of FGR 23,24.

Regarding the vascular indices measured, there was an inverse correlation between UtA PI and placental VI and VFI. Furthermore, the presence of bilateral notching was correlated with a lower placental volume for both gestational age (OEPVR-GA) and estimated fetal weight (OEPVR-FW) as well as lower VIs. This correlation has not been previously reported for FGR fetuses. However, previous studies conducted on normal pregnancies have identified a correlation between first trimester placental VI and UtA Doppler status in the second trimester of pregnancy 25,26. In addition, there is some evidence that in fetuses exhibiting FGR, artery uterine Doppler results are associated with histological evidence of placental underperfusion 27. These findings suggest that VI represents a relationship between abnormal UtA Doppler findings and placental histologic lesions related to poor maternal vascular development and decreased placental perfusion. This relationship suggests that placental VI is related to maternal perfusion of the placenta and directly reflects the deficient trophoblastic invasion present in most cases of FGR. Future studies should focus on the investigation of placental histology and blood flow in relation to 3D power Doppler findings.

### Relationship between placental parameters and 2D Doppler results of UAs in pregnancies with FGR

The present study found no correlation between 3DUS placental parameters and UA Doppler findings. However, other authors who have investigated this potential relationship have reported different results. For example, Luria et al. 13 evaluated 20 FGR fetuses and detected a correlation between VI, FI, and VFI and umbilical PI (r=0.63, r=0.32 and r=0.66, respectively). Further, Guiot et al. 11 evaluated 15 FGR fetuses and observed that only FI was reduced in cases where UA had no end-diastolic flow; in Guiots study, the relationship between FI and PI was not evaluated. Because these studies 11,13 were performed using a vascular placental biopsy technique, it is possible that the observed correlations were due to local placental vascularization, as both studies reported high variability among the samples tested.

Another possible explanation for the lack of agreement with our results is that we included severe growth-restricted fetuses in our investigations. These fetuses were associated with compromised placentas, even when umbilical Doppler findings were normal. Parra-Saavedra et al. 27,28 evaluated placental histology in FGR pregnancies with normal UA Doppler findings and found low placental weight and histological changes secondary to maternal hypoperfusion. These findings suggest that decreased placental volume and the presence of placental vascular lesions are associated with FGR pathophysiological processes even when they are not associated with abnormal UA Doppler results.

### Placental volume in growth-restricted fetuses compared to that in the normal population

In our population of severe growth-restricted fetuses, placental volume was significantly decreased when compared to the general population. This finding is in agreement with findings reported by Pomorski 14, who observed reduced placental volumes in pregnancies with FGR between 22 and 42 weeks of gestation. Additionally, Ulkumen 15 reported decreased placental volumes with FGR between 24 and 40 weeks. It is important to note that the observed-to-expected ratio of placental volume to fetal weight was also decreased, demonstrating that a reduction in placental volume is more important than a reduction in fetal weight.

### Placental vascular indices in growth-restricted fetuses compared to those in a normal population

In severe growth-restricted fetuses, all placental vascular indices (VI, FI and VFI) were significantly reduced when compared to the nomogram described by De Paula et al. 7. These findings align with those from previously reported studies that were performed on less severe growth-restricted fetuses 11-14. An important feature of our study was the use of entire placental 3D evaluations instead of the placental vascular biopsy technique. The placental vascular biopsy technique may not represent the entire placental vascularization because there is well-established variation among samples from different placental regions 11,13,28-30. Thus, evaluation of the entire placenta may better represent its total vascularization.

### Limitations

There are known limitations associated with the 3D power Doppler technique. For example, all vascular parameters are significantly influenced by power Doppler device settings (gain, pulse repetition frequency and depth) 31-34. For this reason, all sonographic parameters were pre-established and were not altered between evaluations. In all our evaluations, we maintained the same distance limit, with just one evaluation detecting a placenta deeper than 55 mm. In our study, there was no correlation between depth and assessed placental parameters. This finding is in agreement with findings previously described by other authors 7,11,12,35.

Another important point pertains to whether placental vascular indices assessed by 3DUS correspond to actual placental vascularization. Some experimental models have provided evidence that placental vascular indices evaluated by US3D correlate with blood vessels and placenta blood flow 36. Furthermore, there is a correlation between decreased 3DUS placental vascular indices and vascular injury 37. However, even if our findings and other studies show that placental vascular indices are significantly lower in growth-restricted fetuses compared to normal fetuses 4,38-40, studies correlating vascular parameters identified by 3DUS to histopathological findings are still required.

In our study, we did not evaluate intra- and inter-observer variabilities. However, previous studies using the same technique and machine settings have demonstrated good repeatability when measuring placental volumes and vascularization by 3DUS 6,7,14,35.

Our study was also limited by the small number of patients evaluated. Because severe FGR is an uncommon complication of different maternal diseases, it would be necessary to perform this study in multiple centers over an extended period to achieve higher enrollment numbers. This limitation represents the clinical challenges of everyday medical practice. Previous studies on this subject describe an even smaller number of cases 11-14.

Our population is heterogeneous in relation to UA Doppler patterns. However, this variability is desirable because it reflects different types and degrees of placental damage.

Additional studies involving a larger numbers of cases could corroborate the hypothesis that 3D placental vascular indices correlate with poor placental implantation and may be related to neonatal outcome.

The present study evaluated, for the first time, the relationship between placental vascular indices and maternal and fetal Doppler findings in severe FGR pregnancies. The results indicated that VI and placental volume are decreased when UtA flow is impaired. This finding may represent a small step toward the elucidation of FGR physiology. Further studies evaluating placental histology and multicentric studies enrolling more patients are required to further investigate this phenomenon.

## AUTHOR CONTRIBUTIONS

Abulé RM performed data analysis and wrote the manuscript. Bernardes LS performed data analysis, wrote and reviewed the manuscript, and was responsible for managing the project. Doro GF performed data analysis and wrote the manuscript. Miyadahira S and Pulcinelli RVF reviewed the manuscript

## Figures and Tables

**Table 1:** Maternal and fetal characteristics of the study group (27 patients).

Maternal and fetal characteristics	n (%) or mean (±SD)
Maternal age (years)	29.11±7.17
Parity	
Primipara [n (%)]	11 (40.7%)
Multipara [n (%)]	16 (59.3%)
Systemic disease [n (%)]	27 (100%)
Hypertensive disorders [n (%)]	25 (92.6%)
GA at evaluation (weeks)	31.3±3.98
EFW at evaluation (grams)	1197±494
Umbilical PI	1.65 (0.80–9.52)
Uterine arteries mean PI	1.6 (0.9–3.14)
Bilateral protodiastolic notch n (%)	13 (48.1%)

* Data are reported as the mean (±standard deviation) and median (range) for continuous variables and as the observed number (percentage) for categorical variables.

GA: gestational age; EFW: estimated fetal weight; PI: pulsatility index.

**Table 2:** Placental volume and vascular indices..

Placental parameters	Median (min–max)
Volume (cm^3^)	146.44 (58.07–529.69)
OEPVR-GA	0.47 (0.25–1.36)
OEPVR-FW	0.72 (0.41–2.06)
VI (%)	8.42 (1.37–26.87)
FI	35.50 (±0.88) ^a^
VFI (%)	2.77 (0.45–10.10)

OEPVR-GA: observed-to-expected placental volume ratio for gestational age; OEPVR-FW: observed-to-expected placental volume ratio for fetal weight; VI: vascularization index; FI: flow index; VFI: vascularization and flow index.

a Mean (±standard deviation).

**Table 3:** Correlation analysis of placental parameters and gestational age..

Placental parameters	*r*	*p*
Volume	0.568	0.002
OEPVR-GA	0.288	0.145
OEPVR-FW	0.150	0.455
VI	0.121	0.548
FI	0.235	0.238
VFI	0.185	0.357

OEPVR-GA: observed-to-expected placental volume ratio for gestational age; OEPVR-FW: observed-to-expected placental volume ratio for fetal weight; VI: vascularization index; FI: flow index; VFI: vascularization and flow index.

**Table 4:** Frequencies of normal and decreased placental parameters classified according to De Paula normal reference ranges 6,7.

Placental parameters	Normal n (%)	Reduced n (%)
Volume for gestational age	12 (44.4%)	15 (55.6%)
Volume for fetal weight	17 (63%)	10 (37%)
VI	15 (55.6%)	12 (44.4%)
FI	13 (48.1%)	14 (51.9%)
VFI	8 (29.6%)	19 (70.4%)

VI: vascularization index; FI: flow index; VFI: vascularization index and flow.

* Statistically significant difference.

**Table 5:** Correlation between uterine and umbilical artery pulsatility index and placental parameters.

Placental parameters	Uterine artery mean PI		Umbilical artery PI	
	*r*	*p*	*r*	*p*
OEPVR-GA	−0.461	0.018*	−0.703	0.722
OEPVR-FW	−0.387	0.051	−0.054	0.793
VI	−0.401	0.042*	0.189	0.355
FI	−0.142	0.490	0.088	0.671
VFI	−0.421	0.048*	0.212	0.297

PI: pulsatility index; OEPVR-GA: observed-to-expected placental volume ratio for gestational age; OEPVR-FW: observed-to-expected placental volume ratio for fetal weight; VI: vascularization index; FI: flow index; VFI: vascularization and flow index.

* Statistically significant difference.

**Table 6:** Comparison of placental parameters according to the presence of bilateral uterine artery diastolic notching.

Placental parameters	No bilateral protodiastolic notch Median (Min-Max) – 14 cases	Bilateral protodiastolic notches Median (Min-Max) – 13 cases	*p*
OEPVR-GA	0.58 (0.33–1.36)	0.36 (0.25–0.75)	0.014 *
OEPVR-FW	0.96 (0.51- 2.06)	0.56 (0.41–1.23)	0.02 *
VI	9.43 (3.33–22.85)	5.96 (1.37–26.87)	0.044 *
FI	35,12 (SD 3.85)	35.49 (SD 5.39)	0.845
IVF	3.23 (1.17–9.23)	2.51 (0.44–10.10)	0.081

OEPVR-GA: observed-to-expected placental volume ratio for gestational age; OEPVR-FW: observed-to-expected placental volume ratio for fetal weight; VI: vascularization index; FI: flow index; VFI: vascularization and flow index.

* Statistically significant difference.
